# Flower extract of *Caragana sinica*. ameliorates DSS-induced ulcerative colitis by affecting TLR4/NF-*κ*B and TLR4/MAPK signaling pathway in a mouse model

**DOI:** 10.22038/IJBMS.2021.53847.12106

**Published:** 2021-05

**Authors:** Ting Li, Qiu-ping Zou, Feng Huang, Gui-guang Cheng, Ze-wei Mao, Ting Wang, Fa-wu Dong, Bao-jing Li, Hong-ping He, Yan-ping Li

**Affiliations:** 1 College of Chinese Medicine, Yunnan University of Chinese Medicine, Kunming, 650500, People’s Republic of China; 2 Yunnan Institute of Food Safety, Kunming University of Science and Technology, Kunming, 650500, People’s Republic of China

**Keywords:** Flower extract of Caragana – sinica, MAPKs, NF-κB, TLR4, Ulcerative colitis

## Abstract

**Objective(s):**

This study aimed to find out the protective effects and preliminary mechanisms of the flower extract of *Caragana sinica* (FEC) on dextran sulfate sodium salt (DSS)-induced colitis.

**Materials and Methods:**

The ulcerative colitis models of mice induced by 3% DSS were established and treated with FEC. Body weight changes, disease activity index (DAI), colon histopathological score, anti-oxidant ability, and the level of inflammatory cytokines were determined. The expression of Toll-like receptor 4 (TLR4) and myeloid differentiation factor 88 (MyD88) were assessed in colonic tissue by immunohistochemical staining. Western blot was used to analyze the expression of TLR4/ nuclear factor kappa-B (NF-*κ*B) and TLR4/ mitogen-activated protein kinase (MAPK) signaling pathway-related proteins.

**Results:**

FEC significantly prevented body weight loss and colonic shortening and reduced the disease activity index and histopathological score (*P*<0.05). Moreover, FEC treatment remarkably down-regulated the levels of myeloperoxidase (MPO), interleukin-1beta (IL-1*β*), tumor necrosis factor-alpha (TNF-*α*), and interleukin 6 (IL-6) and up-regulated the levels of superoxide dismutase (SOD), catalase (CAT), glutathione (GSH), and interleukin 10 (IL-10) in the colon of DSS mice (*P*<0.05). Furthermore, the expression of TLR4/NF-*κ*B and TLR4/MAPK pathway-related proteins was inhibited by FEC (*P*<0.05).

**Conclusion:**

Our findings demonstrated that FEC could serve as a potential therapeutic agent for treatment of ulcerative colitis.

## Introduction

Ulcerative colitis is a chronic inflammatory process of the colon characterized by colonic mucosal and submucosal inflammation, beginning in the rectum and extending proximally in the colon. Its pathological manifestations are diverse, including abdominal pain, diarrhea, and tenesmus, *etc* (1). UC not only affects the life of patients but also significantly increases the risk of colorectal cancer. At present, the pathogenesis of ulcerative colitis has not been fully elucidated. It is implicated that ulcerative colitis might be caused by genetic factors, intestinal environment, immune response, apoptosis, infection, and other factors (2 -4). The main therapeutic drugs are aminosalicylic acid, corticosteroids, immunosuppressants, etc (5, 6). Although sulfasalazine and mesalamine have been used as the first-line medicine for UC therapy, some research has shown that sulfasalazine may have side effects of inhibiting the hematopoietic system, and mesalamine could cause diarrhea (7). Active and mild-to-moderate UC could be treated with corticosteroids and other drugs. However, patients with oral corticosteroids (OCS) had a high prevalence of complications, including difficulty sleeping and weight gain (8, 9). All of these side effects reduce the patient’s quality of life. Therefore, it is necessary to explore new effective medicines with fewer side effects.

Traditional Chinese medicine has long been used in China and clinical applications in recent years show that it has significant advantages in treating ulcerative colitis including low recurrence rate, toxicity, and price. *Caragana sinica* (Buc’hoz) Rehd, widely distributed in China, belongs to the genus *Caragana* of the family Leguminosae (10). Some species of the genus *Caragana* have been used in Chinese traditional medicine for treatment of asthenia syndrome, vascular hypertension, leucorrhagia, neuralgia, rheumatism, arthritis, bruises, and contused wounds (11). The roots of *C. sinica* are usually used for treatment of cough, arthritis, hepatitis, edema, hypertension, *etc* (10). Yellow flowers of *C. sinica* are used as a kind of vegetable in cooking eggs, meats, and soups, and as a remedy for dehydration, indigestion, hypertension, dizziness, tinnitus, and cough in some provinces of China, such as Jiangsu, Shandong, and Hebei (12). Some minority races in Yunnan province of China such as the Bai, Yi, and Miao, have the tradition of eating *C. sinica* flowers (12). It has been reported that the root extract of *C. sinica* has apoptotic, phytoestrogenic, neuroprotective, anti-bacterial, anti-oxidant, and anti-inflammatory effects (13). kobophenol A, a biologically active natural compound isolated from the roots of *C. sinica*, can suppress the expression of inflammatory mediators and cytokines by inhibiting NF-*κ*B nuclear translocation in LPS-stimulated J774 A.1 macrophages (14). However, there are few references on the activities of *C. sinica* flowers, which are also known to be a delicious and healthy functional food in China.

In the present study, we used a simple model of chemically induced acute colitis caused by dextran sulfate sodium (DSS) that is similar to ulcerative colitis (15), in order to evaluate the effects of flower extract of *C. sinica* on colitis and elucidate the underlying mechanism.

## Materials and Methods

***Chemicals and reagent***

DSS (molecular weight 36,000–50,000 Da) was purchased from MP Biomedicals, LLC. Myeloperoxidase (MPO), catalase (CAT), superoxide dismutase (SOD), glutathione peroxidase (GSH), BCA activity assay kit, HRP, and diaminobenzidine were purchased from Nanjing Jiancheng Bioengineering Institute (Nanjing, China). ELISA kits for TNF-*α*, IL-1*β*, IL-6, and IL-10 were purchased from Multi Sciences (Hangzhou, China). Primary antibodies against *β*-actin, NF-*κ*B p65, p-NF-*κ*B p65, I*κ*B*α*, p-I*κ*B*α*, p38, and p-p38 were purchased from Company ABclonal, Inc. (Wuhan, China). Primary antibodies against ERK, p-ERK, JNK, and p-JNK were purchased from Biogot technology, Co, Ltd (Nanjing, China). Primary antibodies against TLR4 and MyD88 were purchased from Wuhan Servicebio Technology Co, Ltd (Wuhan, China). An enhanced chemiluminescent (ECL) plus reagent kit was purchased from Proteintech Group, Inc. (Chicago, USA)

***Preparation of FEC***

Flowers of *C. sinica* (Buc’hoz) Rehd. were purchased from Guilin Pharmaceuticals (Guilin, China) and identified by Dr. Bao-jing Li. A voucher specimen (FEC20180920) was deposited at the Laboratory of Phytochemistry, College of Chinese Medicine, Yunnan University of Chinese Medicine. Flowers of *C. sinica* were extracted three times with one liter of 95% ethanol in a Soxhlet apparatus (Udhna, Surat, Gujarat, India). The liquid was evaporated on a rotavapor (RE-2000A, Shanghai, China) at 65 °C. FEC was finally dried by using a vacuum freeze drier (SCIENTZ-18N, Shanghai, China).

***Extraction and isolation of FEC***

The flowers of *C. sinica* (1 kg) were extracted with 95% ethanol (3 × 5 l) at room temperature (24 hr ×3). The filtrate was evaporated and extracted with EtOAc. The EtOAc part (50 g) was purified on a silica gel column, eluted with chloroform-methanol (CHCl_3_:Me_2_CO) (1:0, 9:1, 8:2, 7:3, 6:4, 5:5, 0:1) to afford seven fractions: Fr. A (3.5 g), Fr. B (7 g), Fr. C (3.5 g), Fr. D(10 g), Fr. E(3 g), Fr. F (3 g), and Fr. G (8.8 g). Fr. A (3.5 g) produced a large amount of white crystals to get compound **6** (0.3 g) after repeated recrystallization in methanol. Fr. B (9:1 part, 7 g) was further separated by silica gel column chromatography with chloroform-methanol (9:1) as an eluent to get Fr. B-1 (0.9 g), Fr. B-2 (0.25 g), Fr. B-3 (1.2 g), Fr. B-4 (0.9 g) and Fr. B-5 (0.76 g). Fr. B-2 was separated by Sephadex LH-20 chromatography (chloroform-methanol 1:1 elution) to obtain compound **1 **(3 mg). Fr. B-3 (1.2 g) was purified on the MCI column, eluted with methanol-water (MeOH-H_2_O) (10%, 30%, 50%, 70%, 90%, and 100%). The 50% part was followed by preparative HPLC (Rp-18, flow rate 5 mL/min, UV detector wavelength 254 nm) and eluted with methanol-water (MeOH-H_2_O) (45%-50%: 0-20 min, 50%-70%: 20-45 min, 70%-80%: 45-55 min) to receive compound **2** (2 mg), compound **4** (3.6 mg), and compound **3** (2.2 mg) at retention times 11.2, 28.9 and 35.7 min, respectively. Compound **7** (8 mg) finally yielded as a white crystal from the 90% part. Fr. C part (3.5 g) was subjected to RP-18 chromatography and eluted with MeOH-H_2_O (10%, 30%, 50%, 70%, 90%, and 100%) to get compound **5** (17.5 mg) from the 50% fraction. 

***Determination of total flavonoid and total phenolic contents of FEC***

The total flavonoid contents (TFCs) were measured using the NaNO_2_-Al (NO_3_)_3_-NaOH’s colorimetric method which was described by a previous study (16). The calibration curve was drawn with different concentrations of rutin as the standard substance. The results were expressed as milligrams of rutin equivalents per gram dry weight of extracts (mg RE/g DW). The rutin calibration curve equation is: Y= 13.156X+0.0082, R^2^= 0.9996.

Total phenolic contents (TPCs) were determined by using Folin-Ciocalteu’s colorimetric method (17). The calibration curve was drawn with gallic acid of different concentrations as the standard substance. The results were expressed as milligrams of gallic acid equivalents per gram dry weight of extracts (mg GAE/g DW). The gallic acid calibration curve equation is: Y= 3.3057X+0.0184, R^2^= 0.9918.

***Animals***

Male C57BL/6 mice (SCXK (Hunan) 2015-030), weighing 20 ± 2 g, were purchased from Kunming Chushang Technology Co, Ltd (Kunming, China) and housed in the experimental animal center under pathogen-free conditions with free access to standard laboratory tap water and chow in a 12-hr light-dark cycle at controlled temperature of 22 ± 2 °C. The animals received humane care and experimental procedures were performed in accordance with the health and care of experimental animals’ guidelines of the Yunnan University of Chinese Medicine (Kunming, China). 

***Establishment of DSS-induced colitis***

After adaptive feeding for at least one week, mice were randomly divided into 4 groups (n =10): Control group, DSS group, high dose of FEC (FEC-H) treatment group (500 mg/kg), and low dose of FEC (FEC-L) treatment group (250 mg/kg). Acute colitis was induced by administration of DSS in drinking water (3%, w/v). The mice received either distilled drinking water (control group) or DSS drinking water (model group and FEC treatment groups) for 7 days and were thereafter provided with drinking water for 3 days (15), the FEC treatment groups (250, 500 mg/kg) were gavaged from day 1 to day 10. Finally, the mice were euthanized, and then the mice in each experimental group were dissected and the colon was cut for analysis.

***Evaluation of DSS-induced colitis***

During the test period, colonic damage in each group was measured using a disease activity index (DAI) score. DAI was recorded daily and scored by assessing loss of body weight, presence of gross bleeding, and stool consistency according to the well-established methods (18). Briefly, DAI was summarized by the following parameters: a. body weight loss (0, no loss. 1, 1–5% loss. 2, 6–10% loss. 3,10–20% loss. or 4, over 20% loss), b. diarrhea (0, normal. 2, loose stools. or 4, watery diarrhea) and c. hematochezia (0, no bleeding. 2, slight bleeding. or 4, gross bleeding) (19).

***Histological evaluation***

For all mice in each group, a 0.5-cm sample was selected in the colon (2 cm above the anus). The samples were cleaned with normal saline and fixed with paraformaldehyde. The colons were then paraffin-embedded and subsequently cut into 5 µM sections. Sections were stained with hematoxylin and eosin (H&E) and checked under 200× and 400× magnification to observe the morphological change of the colonic membrane. The extent of inflammation was determined based on inflammation severity, inflammation extent, and crypt damage visualized in H&E-stained sections (20): 0, rare inflammatory cells in the lamina propria. 1, increased numbers of granulocytes in the lamina propria. 2, confluence of inflammatory cells extending into the submucosa. or 3, transmural extension of the inflammatory infiltrate. Crypt damage was scored as follows: 0, intact crypts. 1, loss of the basal one-third. 2, loss of the basal two-thirds. or 3, entire crypt loss.

***Immunohistochemical staining***

Paraffin slides were de-paraffinized, exposed to anti-TLR4 or anti-MyD88 for 16 hr at 4 °C, and probed with horseradish peroxidase (HRP)-conjugated anti-rabbit antibody for 1 hr at room temperature. Peroxidase activity was visualized by addition of substrate-chromogen solution from the HRP kit. After the sections were incubated with diaminobenzidine, the color was visualized.

***Measurement of oxidative stress markers***

The colonic segments were homogenized in cool normal saline (1:9, colon tissue: normal saline). Supernatants were collected by centrifugation at 12,000 rpm for 10 min at 4 °C. The protein concentration was determined by BCA protein assay. The activities of MPO, SOD, CAT, and GSH in the colon supernatants were examined using MPO, SOD, CAT, and GSH determination kits according to the manufacturer’s instructions. The results are expressed as MPO, SOD, CAT, and GSH units of activity per gram of tissue wet weight (21).

***Cytokines analysis by ELISA***

The colonic segments were homogenized in cool normal saline (1:9, colon tissue: normal saline). Supernatants were collected by centrifugation at 12,000 rpm for 10 min at 4 °C. The amounts of inflammatory cytokines (TNF-*α*, IL-1*β*, IL-6, and IL-10) were determined using ELISA kits according to the manufacturer’s protocols. The absorbance value was detected using a microplate reader.

***Western blot analysis***

Frozen colonic tissue samples were homogenized in ice-cold RIPA buffer, supplemented with a cocktail of protease inhibitions. Then, the homogenate was kept on ice for 30 min and centrifuged at 12,000 rpm for 10 min at 4 °C. The protein concentration of the supernatant was quantified using BCA protein assay kits. After mixed with protein loading buffer and boiled for 10 min at 100 °C, the supernatant was used for the western blotting assay. An equal amount of protein was separated by 10% SDS-PAGE and then transferred onto 0.45 µM polyvinylidene fluoride (PVDF) membranes. Membranes were blocked in 5% skimmed milk in tris buffered saline tween (TBST) for 1 hr at room temperature. After washing with TBST three times, membranes were incubated with primary antibodies overnight at 4 °C. The primary antibodies used in experiments were as follows: *β*-actin (1:2000), NF-*κ*B p65 (1:1000), p-NF-*κ*Bp65 (1:600), I*κ*B*α* (1:1000), p-I*κ*B*α* (1:600), p38 (1:1000), p-p38 (1:1000), ERK (1:1000), p-ERK (1:1000), JNK (1:1000) and p-JNK (1:600). After washing four times for 5 min in TBST buffer, membranes were subsequently incubated with secondary antibody (goat anti-rabbit IgG-HRP, 1:3000 dilution) for 1 hr at room temperature. Finally, the protein band was visualized by a chemiluminescent detection system with ECL substrate after washing five times for 5 min in TBST buffer. The integrated density of each relevant protein band was performed using ImageJ and protein level was normalized against *β*-actin level.

***Statistical analysis***

The data are expressed as the means ± standard error of the mean (SEM) from triplicate experiments. SPSS 17.0 (SPSS, Inc., Chicago, IL, USA) was used for significance of difference (*P*<0.05). Differences among different groups were evaluated by one-way analysis of variance (ANOVA) followed by Tukey’s multiple comparison test (GraphPad Prism Software 5.0). *P*-values<0.05 (*P*<0.05) were accepted as significant difference.

## Results

***Structure of compounds isolated from flowers of C. sinica***

**Figure 1:**
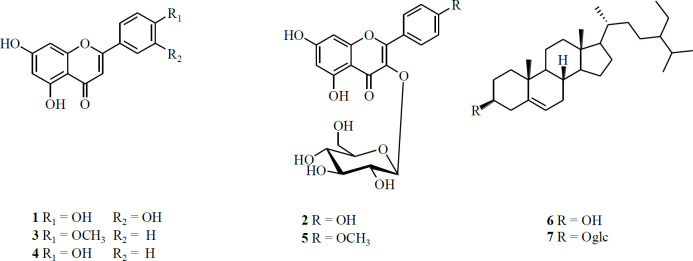
Structures of compounds 1-7 from flower extract of *Caragana *(FEC)

7 compounds, including 5 flavonoids and 2 steroids were isolated from the flowers of *C. sinica*. The structures of these compounds were identified as luteolin (**1**), Kaempferol-3-*O*-*β*-D-glucopyranoside (**2**), Acacetin (**3**), Apigenin (**4**), kaempferide-3-*O*-*β*-D-glucopyranoside (**5**), *β*-sitosterol (**6**), and *β*-daucosterol (**7**) (Figure 1).

***TFCs and TPCs of FEC***

Total flavonoid contents (TFCs) and total polyphenolic contents (TPCs) of FEC were measured. The results demonstrated that the contents of total flavonoids and total polyphenols are relatively high, and the contents of total polyphenols are higher than that of total flavonoids (Table 1).

***Effects of FEC on Weight Loss, colon length reduction and disease activity index (DAI) in UC mice***

**Figure 2:**
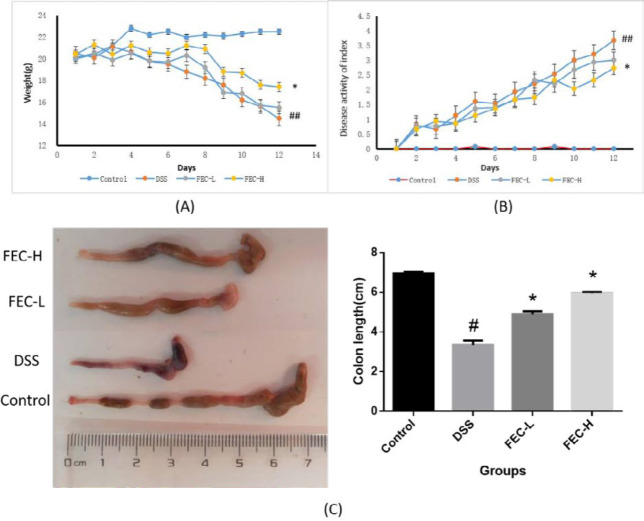
Flavonoids of extract of *Caragana *(FEC) ameliorated the clinical symptoms in mice with DSS-induced acute colitis. Changes in body weight (A), disease activity index (DAI) evaluations in mouse UC models (B), and macroscopic view of the colon and colon length (C) from each group of mice. Data are expressed as means±SD #* P*<0.05, ## *P*<0.01, via a control group. *P<0.05, **P<0.01, via a DSS group

As shown in Figure 2A, body weight loss was insignificant in mice after DSS treatment from day 0 to day 4. However, it became obvious between the control group and other groups after 5 days. Compared with the model group, the FEC-H treatment group had more significant improvement in weight loss than FEC-L (*P*<0.05). And DAI score showed a significant increase from the fourth day of DSS exposure, and the statistical differences were significant compared with the normal group (*P*<0.01). Typical symptoms of clinical colitis such as diarrhea, rectal bleeding, and weight loss in DSS mice were significant from the fourth day. However, the FEC treatment groups showed clearly lower DAI scores than the DSS group in a dose-dependent manner (*P*<0.05) (Figure 2B). At the same time, the FEC treatment group prevented DSS-caused colon length reduction in a mouse UC model in a dose-dependent manner, and the statistical differences were significant compared with the model group (*P*<0.05) (Figure 2C). 

***Effects of FEC on restoration against the colon damage in UC mice***

**Figure 3:**
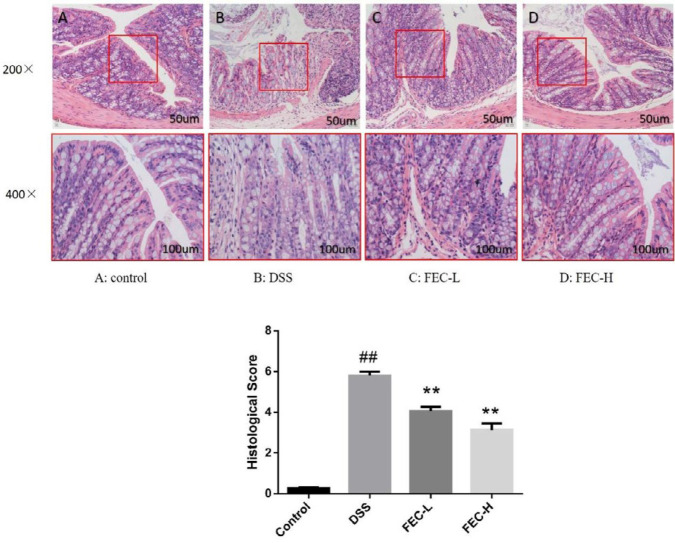
HE staining and histology score of colon tissue in each group (comparison of original magnification × 200 and × 400). A: Control group, B: Model group, C: FEC-L (250 mg/kg), D: FEC-H (500 mg/kg). Data are expressed as the means±SD # *P*<0.05, ## *P*<0.01, via a control group. * *P*<0.05, ** *P*<0.01, via a DSS group

H&E staining showed that the epithelial tissue in the normal group was intact, with obvious crypt and goblet cells and without inflammatory cell invasion; while the epithelial tissue in the model group was seriously damaged, the crypt was deformed, goblet cells were lost and inflammatory cells had infiltrated into the mucosa and submucosa, and the histological score increased significantly (*P*<0.01). In FEC treatment groups, especially in the high-dose group of FEC, we could see that the structures of epithelial tissue and goblet cells were relatively complete, and the degree of inflammatory cells infiltrating into the mucosa and submucosa had been well repaired. As for the FEC-L treatment group, the phenomenon about the recovery of colon tissue was not as good as that of the high-dose group, but it was significantly better than the model group. For histological scores, the statistical differences of both FEC groups were significant compared with the model group (*P*<0.01) (Figure 3).

***Effects of FEC on the anti-oxidant ability of UC model***

**Figure 4:**
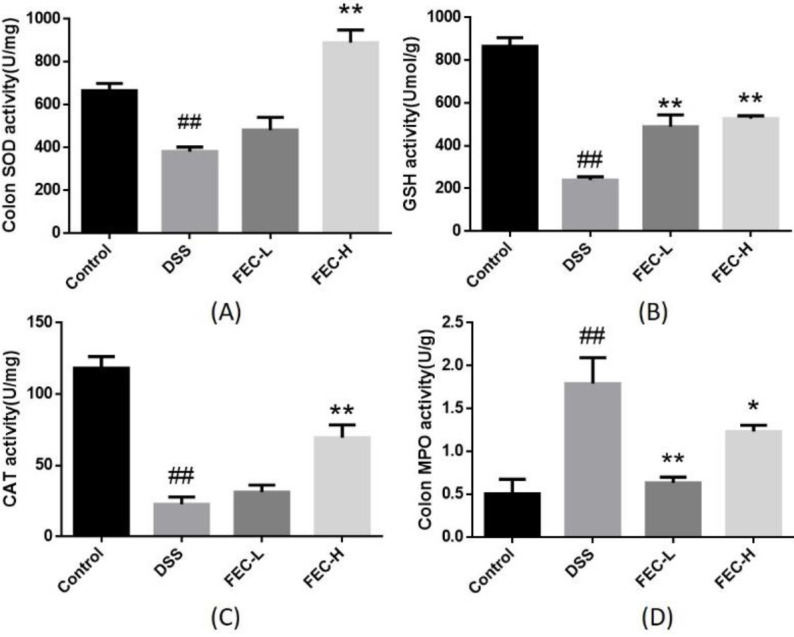
Effects of flavonoids of extract of *Caragana *(FEC) on oxidative biomarkers in a mouse UC model. The effects of FEC on the activity of SOD (A), GSH (B), CAT (C), and MPO (D) in a mouse UC model. Data are expressed as means±SD # *P*<0.05, ## *P*<0.01, via a control group. * *P*<0.05, ** *P*<0.01, via a DSS group

DSS treatment increased oxidative stress of the UC model by reducing the levels SOD (Figure 4A), GSH (Figure 4B), and CAT (Figure 4C), and increasing the level of MPO (Figure 4D) when compared with healthy animals (*P*<0.01). Comparatively, the FEC-H treatment group showed strong anti-oxidant capacity by increasing the levels of SOD (Figure 4A), GSH (Figure 4B), and CAT (Figure 4C), and reducing the level of MPO (Figure 4D) (*P*<0.01). However, in the FEC-L treatment group, the increasing trend of SOD, GSH, and CAT could also be observed, and only the level of GSH increased significantly compared with the model group. It is worth noting that the level of MPO in serum of the low-dose group was reduced more than that of the high-dose group.

***FEC ameliorated inflammatory response in the mouse model of UC***

**Figure 5:**
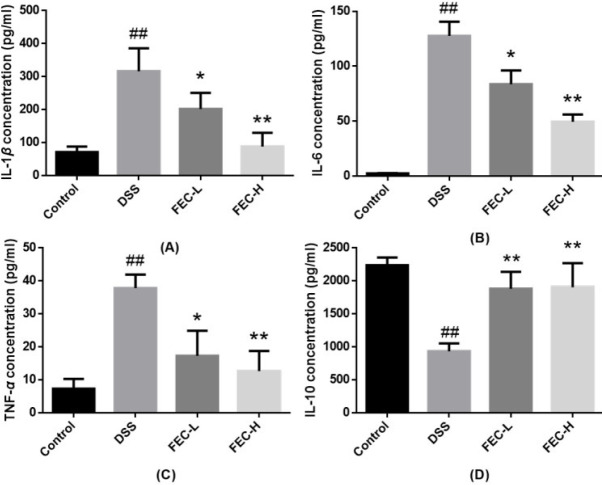
Effects of flavonoids of extract of *Caragana *(FEC) on serum levels of inflammatory factors in a mouse UC model. The effects of FEC on serum levels of IL-1β (A), IL-6 (B), TNF-α (C), and IL-10 (D). Data are expressed as means±SD # *P*<0.05, ## *P*<0.01, via a control group. * *P*<0.05, ** *P*<0.01, via a DSS group

To evaluate the anti-inflammatory effect of FEC, we tested the levels of cytokines IL-1*β*, TNF-*α*, IL-6, and IL-10. ELISA analysis showed that DSS treatment increased the serum levels of inflammatory factors IL-1*β* (Figure 5A), IL-6 (Figure 5B), and TNF-*α* (Figure 5C) and reduced the level of IL-10 (Figure 5D) when compared with the normal group (*P*<0.01). The FEC-H treatment group had inhibited serum levels of inflammatory factors IL-1*β*, IL-6, and TNF-*α* and increased levels of IL-10 better than the FEC-L treatment group when compared with the model group (*P*<0.01).

***Effects of FEC on regulating key molecules involved in NF-*** ***κ*** ***B/MAPK signaling pathway***

**Figure 6:**
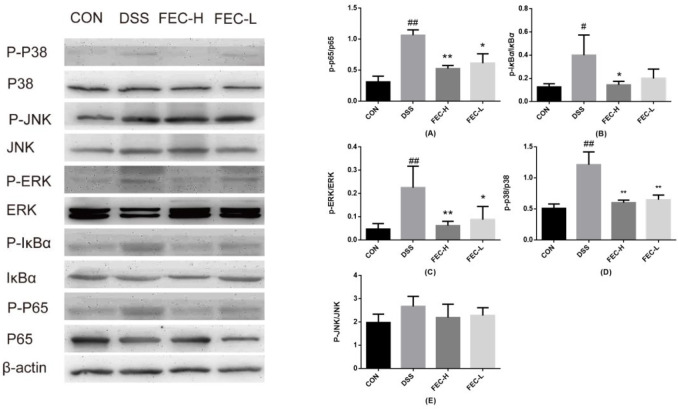
Effects of flavonoids of extract of *Caragana* (FEC) on the levels of key molecules involved in nuclear factor kappa-B (NF-*κ*B) and mitogen-activated protein kinase (MAPK) signaling pathway. Expression of MAPK phosphorylation (p-38, ERK, and JNK) and NF-*κ*B phosphorylation (p-65, I*κ*B*α*) in the colon samples isolated from mice analyzed by Western blot analysis. Data are expressed as means±SD # *P*<0.05, ## *P*<0.01, via a control group. * *P*<0.05, ** *P*<0.01, via a DSS group

 We detected the protein levels of p65 and p-p65 in colon tissue by western blotting analysis. As shown in Figure 6, we found that protein expression of p-p65 increased in the DSS-induced group compared with the control group (*P*<0.01). In contrast, the expression of p-p65 in the FEC-H treatment group was significantly lower than that in the FEC-L treatment group (*P*<0.01). In consideration that the rapid phosphorylation of I*κ*B*α* is the key step for accumulation of p-p65 in the nucleus, the phosphorylation level of I*κ*B*α* was checked. Our results showed that DSS significantly induced the phosphorylation of I*κ*B*α* (*P*<0.05), which was also significantly suppressed by different concentration FEC treatment in colon tissue, surprisingly, the effect of FEC-H was better than that of FEC-L (*P*<0.05) (Figure 6).

MAPKs are some of the important signal transduction molecules regulating cell differentiation and apoptosis (22). Western blotting results in our study showed that the protein levels of p-p38 and p-ERK in the DSS-induced group were significantly higher than those in the control group (*P*<0.01). Although p-JNK also showed an increasing trend, there was no significant difference. Compared with the model group, the FEC-H treatment group had decreased p-p38 and p-ERK protein levels more significantly than the FEC-L treatment group (*P*<0.01). However, there was no significant difference in the downward trend of p-JNK between the FEC treatment group and the model group (Figure 6).

***Effects of FEC treatment on TLR4 and MyD88 activity in the UC model***

**Figure 7:**
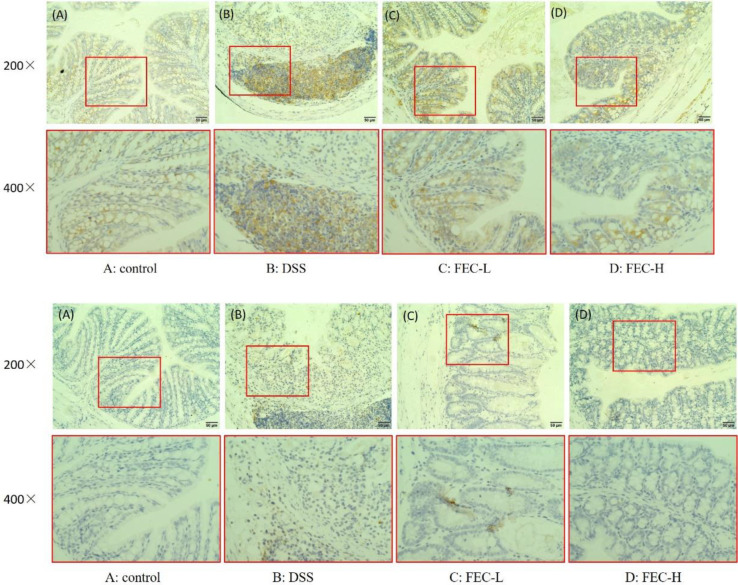
Toll-like receptor 4 (TLR4) and myeloid differentiation factor 88 (MyD88) immunohistochemical staining findings in colon tissue (comparison of original magnification × 200 and × 400). (A): Control group, (B): Model group, (C): FEC-L (250 mg/kg), (D): FEC-H (500 mg/kg). (a): TLR4 immunohistochemical staining, (b): MyD88 immunohistochemical staining

The TLR4/MyD88 signaling pathway plays an important role in inducement of inflammation through regulating the activity of transcription factors. Immunohistochemical staining showed that expression of TLR4 in both epithelial cells and inflammatory cells of colonic tissues from the DSS group was remarkably up-regulated compared with that of the control group. When FEC was given, the expression of TLR4 in colonic sections was notably suppressed. Obviously, the inhibition effect of high dose was much better than that of low dose (Figure 7a). However, the expression of MyD88 in the epithelial cells and inflammatory cells of colon tissue in the FEC treatment group was close to that of the normal group (Figure 7b).

## Discussion

Ulcerative colitis (UC) is a chronic inflammatory disease occurring in the rectum and colon mucosa, which is one of the two main forms of inflammatory bowel disease (IBD). Suffering the disease seriously affects the quality of the patients’ life because of its typical characteristics of recurrent attacks. Previous studies have established DAI, body weight loss, colonic length, and histological scores as the main parameters used to determine its degree (23). So significantly high index of DAI in DSS-induced UC mice in our current study indicated successful establishment of the UC model in mice. And treatment of both high and low dosages of FEC treatment could reduce DAI, improve body weight and colon length, and attenuate histopathological change in immunological UC in mice. 

The symptoms of ulcerative colitis have been characterized as over-secretion of inflammatory cytokines and epithelial barrier dysfunction (24). MPO is an enzyme mainly detected in neutrophils, and its activity reflects the degree of neutrophil infiltration, so it can be used as a marker of acute inflammation (25). In the present study, the significantly reduced level of MPO even in the low dosage of FEC group indicated a remarkable improvement effect in acute inflammation. We also showed that FEC treatment inhibits the release of pro-inflammatory cytokines such as IL-1*β*, TNF-*α*, and IL-6 and obviously accelerates the expression level of IL-10 in a dose-dependent manner. These data clearly demonstrated that FEC alleviated the symptoms of DSS-induced colitis, improved the disease histopathology, and implied a protective role for FEC in colitis.

Among the immune regulatory factors, oxidative stress has been proposed as one of the major mechanisms involved in the pathophysiology of IBD (26, 27). It has been confirmed that chronic intestinal inflammation was associated with excessive production of reactive oxygen (ROS), leading to oxidative stress, which has been implicated in several human diseases including IBD (28, 29). In fact, in IBD, oxidative damage can be evaluated by investigating either of the oxidant or anti-oxidant markers, such as SOD, CAT, GPX, GSH, vitamins, and so on (30-32). GSH plays a vital role in protecting the intestinal cells and as a defense mechanism against inflammation (33). And it was reported that DSS treatment can significantly reduce SOD and CAT levels and cause tissue damage similar to that of human IBD (34). Our study showed that the FEC treatment group could inhibit colitis by regulating anti-oxidant mediators and inhibiting SOD, CAT, and GSH productions, which means FEC might exhibit anti-UC activity through anti-oxidant activity, and the anti-oxidant activity was better when a higher dose of FEC was given.

TLR4 plays a critical role in the process of inflammation (35). Numerous studies have shown that TLR4 overexpresses in colon mucosa of colorectal cancer patients, but TLR4 knockout mice can significantly reduce the incidence rate of colon cancer (36). In addition, increasing numerous research also has shown that Toll-like receptors recognize the structural elements of microorganisms, leading to nuclear transcription of NF-*κ*B and activation of MAPK family members (37). The processes of TLR4/NF- *κ*B/MAPKs activation need several key links. MyD88 is an important downstream key link of TLR4 in the TLR signaling pathway. Therefore, we tested the expressions of TLR4 and MyD88 proteins in colonic mucosa by the immunohistochemical staining method and the results revealed that the level of TLR4 protein in model group mice was highly expressed while it was significantly reduced in the FEC-H treatment group compared with FEC-L. Of note, no significant difference in the protein expression levels of MyD88 between the control group and model group was observed, which indicated that other pathways may also play an important role in the TLR4/NF-*κ*B/MAPKs signaling pathway. However, it is worth further research to elucidate a more precise mechanism.

He *et al*. confirm that up-regulation of TLR4 and NF-*κ*B were significantly reversed by alpinetin treatment on DSS-induced colitis in mice (38); and Choi *et al.* found that Isoliquiritigenin treatment attenuated colitis via suppressing the phosphorylation of MAPKs and activation of NK-*κ*B in colon tissue (39). To explore the exact molecular mechanism of TLR4 downstream for the function of FEC, the effects of each component and its combination on the NF-*κ*B and MAPK related molecules in colon tissue of mice with UC were investigated. The results of our study showed that the FEC treatment group inhibited NF-*κ*B P65 phosphorylation and I*κ*B*α* degradation in a dose-dependent manner. This means that FEC treatment could suppress the activation of signal transduction to the I*κ*B kinase (IKK) complex, and then inhibit the releasing and binding of NF-*κ*B to the promoter of the target gene. The activated NF-*κ*B by entering the nucleus and inducing expression of numerous genes involved in innate and adaptive immune regulation, inflammatory responses, and anti-apoptotic mechanisms were suppressed by FEC treatment (40,-42).

MAPKs are a class of serine/threonine protein kinases in cells involved in the regulation of key cellular processes such as gene induction, cell survival/apoptosis, proliferation, differentiation, cellular stress, and inflammatory responses (43). At present, it is found that there are multiple parallel signal pathways such as the p38 MAPK pathway, ERK1 / 2 pathway, and JNK pathway. p38MAPK affects the process of inflammatory response and the balance of inflammation and anti-inflammatory factors by regulating the production of proinflammatory cytokines TNF-*α*, IL-1*β*, IL-6, IL-8, and IL-10. Also, p38 can positively regulate the activity of NF-*κ*B and finally participate in the release of cytokines and inflammatory mediators (44, 45). Our results indicated that FEC pretreatment significantly suppressed phosphorylation of P38 and ERK and may thus inhibit the production of IL-1*β*, IL-6, and TNF-*α*, showing an anti-inflammatory activity. In addition, high dose of FEC showed stronger inhibitory activity. However, the effect of FEC pretreatment on inhibiting the phosphorylation of JNK is indeed not significant, which may mean that FEC mainly regulates the production of pro-inflammatory cytokines and anti-inflammatory factors through ERK and P38 pathways in the MAPKs pathway to affect the process of inflammation.

*C. sinica*, a deciduous shrub, is widely distributed in Asia, particularly in Korea, Japan, and China (10). Previous studies have focused on the chemical constituents and bioactivity capacity of *C. sinica* roots. Min *et al*. found that *C. sinica* root extracts significantly suppressed IL-1*β*-stimulation of MAPKs, NF-*κ*B signaling pathway (46). A study showed that kobophenol A from roots of *C. Sinica *suppressed pro-inflammatory mediators by blocking the nuclear translocation of NF-*κ*B in LPS-stimulated J774 A.1 cells (14). In this paper, the efficacy of FEC in the treatment of ulcers was evaluated by in *vivo* methods. At the same time, with the aim of finding anti-inflammatory compounds, 7 compounds including 5 flavonoids were isolated from the flowers of *C. sinica*. Of these compounds, luteolin was reported to have an anti-inflammatory effect through p38/MK2/TTP-regulated mRNA stability and anti-oxidative effects through its excellent radical scavenging and cytoprotective properties (47, 48). Astragalin (kaempferol-3-*O*-*β*-D-glucoside) exerted an anti-inflammatory effect through NF-*κ*B pathway inhibition and attenuated murine colitis and can be used as a potential therapeutic agent for IBD (49). Apigenin has been demonstrated to have anti-inflammatory and anti-oxidative effects and can be used in TNBS and DSS colitis models of IBD (50). Another compound acacetin has been considered to have anti-inflammatory and anti-oxidative effects in inhibiting sepsis-induced acute lung injury model (51). From the above results and related literature, we can infer that the roots and flowers of *C. sinica* may contain common materials that had an anti-inflammatory effect. Therefore, it is reasonable to assume that FEC amelioration of colitis mice could be related to the anti-oxidative and anti-inflammatory effects of flavonoids. 

**Table 1:** Total polyphenols and flavonoids of extract of *Caragana *(FEC)

**Sample**	**Total flavonoids ** **mg RE/100 g DW**	**Total polyphenols ** **mg GAE/100 g DW**
FEC	47.90 ± 0.37	195.13 ± 0.64

* Results are expressed as mean±standard deviation

## Conclusion

Our work confirmed FEC could inhibit colitis by regulating anti-oxidant mediators and inhibiting MPO, SOD, CAT and GSH production, inducible IL-1*β*, IL-6, and TNF-*α* production and inhibit activation and nuclear translocation of p65, I*κ*B subunits of nuclear factor-kappa B, and suppress phosphorylation of MAPKs-related proteins by reducing the expression of TLR4 at the protein level. Therefore, our study ultimately suggested that FEC is an effective anti-inflammatory and anti-oxidant supplement, which may be a promising therapeutic option for UC. 
